# Supplementary material for: The validation of the Beijing version of the Montreal Cognitive Assessment in Chinese patients undergoing hemodialysis
Source: PLoS One. 2020 Jan 9;15(1):e0227073. doi: 10.1371/journal.pone.0227073 (PMC6952078; doi:10.1371/journal.pone.0227073)
Supplement: S1 File — (DOCX) [file pone.0227073.s001.docx]

STROBE Statement—checklist of items that should be included in reports of observational studies

|  | Item No. | Recommendation | Page  No. | Relevant text from manuscript |
| --- | --- | --- | --- | --- |
| **Title and abstract** | 1 | (*a*) Indicate the study’s design with a commonly used term in the title or the abstract | 1 | The validation of the Beijing version of the Montreal Cognitive Assessment in Chinese patients undergoing hemodialysis |
|  |  | (*b*) Provide in the abstract an informative and balanced summary of what was done and what was found | 2 | We assessed 613 patients undergoing hemodialysis using the MoCA-BJ, the Mini-Mental State Examination (MMSE), and a comprehensive neuropsychological battery. Cognitive dysfunction was defined by the fifth version of the Diagnostic and Statistical Manual of Mental Disorders (DSM-V). Spearman’s correlation and linear regression were used to estimate the performance of the MoCA-BJ and MMSE in predicting cognitive impairment. A receiver operating characteristic (ROC) curve analysis was used to evaluate the utility of various cutoffs of the MoCA-BJ and MMSE for predicting cognitive impairment. |
| Introduction | | | |  |
| Background/rationale | 2 | Explain the scientific background and rationale for the investigation being reported | 4 | data concerning the predictive value of MoCA in hemodialysis patients were mostly from other ethnic groups and the sample size of the previous studies was relatively small [10, 11]. |
| Objectives | 3 | State specific objectives, including any prespecified hypotheses | 5 | Therefore, we aimed to validate the sensitivity, specificity and predictive value of the MoCA-BJ as a screening tool in detecting cognitive impairment which was determined by a comprehensive neuropsychological battery in a group of Chinese hemodialysis patients. |
| Methods | | | |  |
| Study design | 4 | Present key elements of study design early in the paper | 5 | A validation analysiss was performed on the basis of a prospective cohort study were performed |
| Setting | 5 | Describe the setting, locations, and relevant dates, including periods of recruitment, exposure, follow-up, and data collection | 7 | Spearman’s correlation and linear regression were used to estimate the performance of the MoCA-BJ and MMSE in predicting cognitive impairment. A receiver operating characteristic (ROC) curve analysis was used to evaluate the utility of various cutoffs of the MoCA-BJ and MMSE for predicting cognitive impairment. |
| Participants | 6 | (*a*) *Cohort study*—Give the eligibility criteria, and the sources and methods of selection of participants. Describe methods of follow-up  *Case-control study*—Give the eligibility criteria, and the sources and methods of case ascertainment and control selection. Give the rationale for the choice of cases and controls  *Cross-sectional study*—Give the eligibility criteria, and the sources and methods of selection of participants | 8 | The study was performed using the data repository of the cohort study of CI in Chinese patients undergoing hemodialysis (CODE) (ClinicalTrials.gov ID: NCT03251573), including 613 patients recruited from 11 HD centers in Beijing between April 2017 and June 2017. The eligibility criteria for participants were as follows: (1) aged 50-80 years, (2) had end-stage kidney disease, (3) were treated with long-term outpatient HD for the previous ≥3 months, (4) their HD team agreed to join the investigation and patients were willing to provide written informed consent, (5) ability to complete a 90 min cognitive and physical function battery, and (6) first language is Chinese. |
|  |  | (*b*) *Cohort study*—For matched studies, give matching criteria and number of exposed and unexposed  *Case-control study*—For matched studies, give matching criteria and the number of controls per case |  |  |
| Variables | 7 | Clearly define all outcomes, exposures, predictors, potential confounders, and effect modifiers. Give diagnostic criteria, if applicable | 9 | We assessed 613 patients undergoing hemodialysis using the MoCA-BJ, the Mini-Mental State Examination (MMSE), and a comprehensive neuropsychological battery. Cognitive dysfunction was defined by the fifth version of the Diagnostic and Statistical Manual of Mental Disorders (DSM-V). |
| Data sources/ measurement | 8* | For each variable of interest, give sources of data and details of methods of assessment (measurement). Describe comparability of assessment methods if there is more than one group | *8* |  |
| Bias | 9 | Describe any efforts to address potential sources of bias | 9 |  |
| Study size | 10 | Explain how the study size was arrived at | 7 | We assessed 613 patients undergoing hemodialysis using the MoCA-BJ, the Mini-Mental State Examination (MMSE), and a comprehensive neuropsychological battery. |

Continued on next page

| Quantitative variables | 11 | Explain how quantitative variables were handled in the analyses. If applicable, describe which groupings were chosen and why | 5 | Non cognitive and cognitive impairment |
| --- | --- | --- | --- | --- |
| Statistical methods | 12 | (*a*) Describe all statistical methods, including those used to control for confounding | 9 | Spearman’s correlation and linear regression were used to estimate the performance of the MoCA-BJ and MMSE in predicting cognitive impairment. A receiver operating characteristic (ROC) curve analysis was used to evaluate the utility of various |
|  |  | (*b*) Describe any methods used to examine subgroups and interactions | 9 | N/A |
|  |  | (*c*) Explain how missing data were addressed |  |  |
|  |  | (*d*) *Cohort study*—If applicable, explain how loss to follow-up was addressed  *Case-control study*—If applicable, explain how matching of cases and controls was addressed  *Cross-sectional study*—If applicable, describe analytical methods taking account of sampling strategy |  |  |
|  |  | (*e*) Describe any sensitivity analyses | 7 | N/A |
| Results | | | | |
| Participants | 13* | (a) Report numbers of individuals at each stage of study—eg numbers potentially eligible, examined for eligibility, confirmed eligible, included in the study, completing follow-up, and analysed | 10 |  |
|  |  | (b) Give reasons for non-participation at each stage | 10 |  |
|  |  | (c) Consider use of a flow diagram | 11 |  |
| Descriptive data | 14* | (a) Give characteristics of study participants (eg demographic, clinical, social) and information on exposures and potential confounders | 11 |  |
|  |  | (b) Indicate number of participants with missing data for each variable of interest | 12 |  |
|  |  | (c) *Cohort study*—Summarise follow-up time (eg, average and total amount) | 12 |  |
| Outcome data | 15* | *Cohort study*—Report numbers of outcome events or summary measures over time | *12* |  |
|  |  | *Case-control study—*Report numbers in each exposure category, or summary measures of exposure |  |  |
|  |  | *Cross-sectional study—*Report numbers of outcome events or summary measures |  |  |
| Main results | 16 | (*a*) Give unadjusted estimates and, if applicable, confounder-adjusted estimates and their precision (eg, 95% confidence interval). Make clear which confounders were adjusted for and why they were included | 12-14 | The ROC analysis identified an optimal cut-off for the MoCA at ≤ 24 points (Table 4), with a sensitivity of 87.7%, a specificity of 75.2 % and an area under the curve of 0.891 (95% CI, 0.859-0.924). In comparison, the MMSE only achieved a sensitivity of 70.4%, and a specificity of 76.9%, and AUC of 0.823 (95% CI, 0.786-0.860) for an optimal cut-off of ≤ 26 points (Table 4, Figure 1). |
|  |  | (*b*) Report category boundaries when continuous variables were categorized |  |  |
|  |  | (*c*) If relevant, consider translating estimates of relative risk into absolute risk for a meaningful time period |  |  |

Continued on next page

| Other analyses | 17 | Report other analyses done—eg analyses of subgroups and interactions, and sensitivity analyses |  |  |
| --- | --- | --- | --- | --- |
| Discussion | | | | |
| Key results | 18 | Summarise key results with reference to study objectives | 15 |  |
| Limitations | 19 | Discuss limitations of the study, taking into account sources of potential bias or imprecision. Discuss both direction and magnitude of any potential bias | 15 |  |
| Interpretation | 20 | Give a cautious overall interpretation of results considering objectives, limitations, multiplicity of analyses, results from similar studies, and other relevant evidence | 16 |  |
| Generalisability | 21 | Discuss the generalisability (external validity) of the study results | 16 |  |
| Other information | |  | | |
| Funding | 22 | Give the source of funding and the role of the funders for the present study and, if applicable, for the original study on which the present article is based | 19 | Funding  This project is supported by a grant from the Beijing Municipal Science & Technology Commission (NO. Z161100002616005) |
| Supporting information | 23 | S1 Table. Basic characteristics of the participants.xlsx | 26 | Provided dataset for the results in this manuscript. |
|  | 24 | S2 Table. Neuropsychological tests of five cognitive domains of the participant.xlsx | 26 | Provided dataset for the results in this manuscript. |

*Give information separately for cases and controls in case-control studies and, if applicable, for exposed and unexposed groups in cohort and cross-sectional studies.

**Note:** An Explanation and Elaboration article discusses each checklist item and gives methodological background and published examples of transparent reporting. The STROBE checklist is best used in conjunction with this article (freely available on the Web sites of PLoS Medicine at http://www.plosmedicine.org/, Annals of Internal Medicine at http://www.annals.org/, and Epidemiology at http://www.epidem.com/). Information on the STROBE Initiative is available at www.strobe-statement.org.
